# A Frequency- and Power-Dependent Semi-Analytical Model for Wideband RF Energy Harvesting Rectifiers

**DOI:** 10.3390/mi17010030

**Published:** 2025-12-26

**Authors:** Sadık Zuhur

**Affiliations:** Department of Electricity and Energy, Igdir University, Igdir 76000, Türkiye; sadik.zuhur@igdir.edu.tr

**Keywords:** RF energy harvesting, wireless power transfer, semi-analytical model, RF-DC conversion, impedance matching

## Abstract

In this study, a new semi-analytical model was developed that can calculate the output voltage of low-power microwave rectifiers as a function of frequency and input power. The model integrates diode rectification characteristics and frequency-dependent impedance mismatches within the same mathematical structure. Defined by second-order polynomial expressions for input power and frequency, the model directly incorporates reflection coefficient (S_11_) data into the equations to account for frequency-dependent power losses caused by impedance mismatch, thereby improving calculation accuracy under wide-band conditions. To validate the model, a wide-band rectifier prototype with an FR4-based T-type matching network and a voltage doubler structure was designed and manufactured. Model calculations showed over 95% agreement with simulation results and closely followed the measured output voltage trends over the 0.5–3 GHz frequency range and input power levels from −12 dBm to 0 dBm. The proposed model provides a design-oriented and computationally efficient tool for wide-band, low-power RF energy harvesting and wireless power transfer applications, enabling rapid evaluation of impedance matching strategies with reduced reliance on electromagnetic simulations.

## 1. Introduction

**Figure 1:**
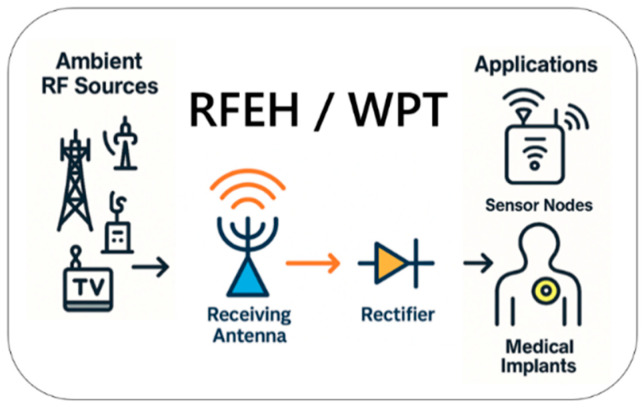
Conceptual illustration of RF energy harvesting (RFEH) and wireless power transfer (WPT) systems from ambient sources to low-power applications.

Micro-energy harvesting technologies have recently emerged as promising solutions for reducing or eliminating battery dependence in low-power electronic devices. These technologies convert ambient energy into usable electrical power for applications such as IoT devices, wireless sensor networks, and self-powered electronic systems [1,2,3,4]. Among the various harvesting approaches reported in the literature, radio-frequency energy harvesting (RFEH) has attracted increasing attention due to the growing abundance of ambient communication signals [5,6,7,8] (Figure 1).

Recent studies on RFEH and microwave Wireless Power Transfer (WPT) systems show that these systems have a multilayer structure consisting of an antenna, an impedance matching network and rectifier circuits, and that the performance of these subsystems can only be accurately evaluated using a holistic approach [9,10]. As emphasized in the system-level analyses reported by [11], realistic modeling requires consideration of both rectification behavior and frequency-dependent reflection losses. Among these subsystems, the rectifier diode plays a central role in determining the non-linear behavior of the RF-to-DC conversion process, particularly under wideband and low input power conditions.

However, many theoretical models in the literature neglect the nonlinear dependence of rectification behavior on frequency and input power [12,13]. Small-signal models based on constant impedance assumptions fail to capture rectification behavior that varies with frequency and input power, particularly under low-power operating conditions [14]. In real-world operating environments, however, the input power is often not constant and can fluctuate significantly over time; the sensitivity of the diode and rectifier impedance to these variations directly affects the overall performance of the circuit [15]. While detailed models that represent these interactions more realistically have been proposed [16], most of these models rely on complex mathematical structures, complicating the design process.

Recent studies aimed at improving the performance of wide-band RF energy harvesters [17,18,19] generally focus on specific frequency ranges or fixed input powers, a model approach sensitive to frequency and power variations remains lacking. Therefore, a model that integrates both diode rectification characteristics and frequency-dependent impedance mismatches within the same mathematical formulation would fill an important gap in the analysis and design of wide-band rectifier circuits.

To address this need, this study proposes a new semi-analytical model based on experimentally verifiable parameters that provides high-accuracy calculations for wide-band applications. The proposed semi-analytical model focuses on the rectification behavior of the diode while incorporating the effects of the remaining circuit elements in a cumulative and frequency-dependent manner. The model is expressed using second-order polynomials as functions of input power and frequency, and it explicitly incorporates the reflection coefficient (S_11_) to account for power losses arising from impedance mismatches. The proposed model demonstrates more than 95% agreement with simulation results and shows close correspondence with experimental measurements. The accuracy of the model is validated through the fabrication of a compact prototype employing an FR4-based T-type matching network and a voltage-doubling rectifier structure, confirming its suitability for both theoretical analysis and practical circuit design.

The remainder of this article is organized as follows: Section 2 introduces the proposed semi-analytical model, Section 3 describes the prototype used for model validation, Section 4 presents the experimental validation results, and Section 5 concludes the paper with a discussion of the main findings and directions for future work.

## 2. Proposed Semi-Analytical Model

The semi-analytical model presented in this study was developed to calculate the output voltage of the rectifier circuit in RFEH and WPT systems as a function of input frequency (f) and input power level (P). The model is suitable for use in wideband applications and is supported by circuit simulations. The proposed model is valid for input power levels between –12 dBm and 0 dBm and for the frequency range of 0.5–3 GHz, for which the regression coefficients were derived from simulation data.

### 2.1. Large-Signal Diode Rectification Model

The rectification characteristics of Schottky diodes operating in the large signal regime vary considerably with the amplitude of the signal (V_p)_ and frequency. The parameters and coefficients used in the proposed model are derived from the results obtained from circuit simulations. The current-voltage relationship of the diode is defined by the Shockley equation, as given in Equation (1):

(1) $$I_{D}\left(t\right)={\;I}_{S}\left(e^{\frac{V_{D}\left(t\right)}{nV_{T}}}-1\right)$$

where I_D_(t) is the time-dependent diode current; I_S_ is the saturation current; n is the ideality factor; V_T_ is the thermal voltage; and V_D_(t) is the time-dependent voltage at the diode terminals. When the input signal is sinusoidal, this voltage is defined as shown in Equation (2):

(2) $$V_{D}\left(t\right)=Vp\;sin\left(2\pi ft\right)$$

This nonlinearity causes the current passing through the diode to contain higher-order harmonics. The rectification behavior is evaluated based on the DC output component at the output and this component is expressed as the time-average of the current as shown in Equation (3):

(3) $$V_{\mathrm{out}}\propto \left\langle I_{D}\left(t\right)\right\rangle =\frac{1}{T}\int I_{D}\left(t\right)\;dt$$

However, since the analytical solution of this integral is not possible, the expression I_D_(t) is expanded using a Taylor series expansion, as presented in Equation (4):

(4) $$I_{D}\left(t\right)\;\approx \;I_{S}\left(\frac{V_{D}\left(t\right)}{nV_{T}}+\frac{1}{2}{\left(\frac{V_{D}\left(t\right)}{nV_{T}}\right)}^{2}+\cdots \right)$$

The rectified output voltage V_out_ is obtained by averaging the diode current over time. Since the input signal is sinusoidal and the average of odd-order terms in the Taylor expansion is zero, only even-order terms contribute to the DC component. Because a closed-form analytical expression could not be obtained for this DC component, the frequency- and input power-dependent behavior of the output voltage was modeled using regression-based polynomial expressions derived from simulation data.

### 2.2. Polynomial Representation of Frequency- and Power-Dependent DC Output

In rectifier circuits, the rectification behavior is frequency-dependent due to the diode junction capacitance and circuit parasitics, which cause the rectified DC component to decrease as the frequency increases. As a result, the output voltage exhibits a frequency-dependent maximum. This behavior is modeled using second- and fourth-order frequency terms, which represent the dominant DC contributions arising from the large-signal rectification process. Accordingly, the frequency-dependent DC component is expressed by the polynomial structure given in Equation (5):

(5) $$V_{out}(f)\;\propto {\;\alpha }_{0}\;+\;\alpha_{2}f^{2}\;+{\;\alpha }_{4}f^{4}$$

Here, f denotes the input frequency, while α_0_, α_2_, and α_4_ are regression coefficients dependent on the input power. Higher even-order terms were also examined during preliminary regression analyses; however, as they resulted in negligible accuracy improvement, only second- and fourth-order terms were retained in the final model.

Since V_p_ cannot be measured directly in practice, this quantity is incorporated into the model by relating it to the input power P. Furthermore, considering the sensitivity of the rectification behavior to frequency (f), frequency-dependent DC contributions are simplified using the regression method. Thus, the relationship between the output voltage and frequency and input power is modeled using Equation (6):

(6) $$V_{out}(f,P)\;=\;A(P)\;(\alpha_{0}(P)\;+{\;\alpha }_{2}(P)\;f^{2}\;+\;\alpha_{4}(P)\;f^{4})\;+\;B(P)$$

Here, A(P), α_i_(P), and B(P) are regression coefficients expressed as second-order polynomials, depending on the input power. The frequency squared (f^2^) and fourth-order (f^4^) terms represent the dominant DC contributions resulting from the nonlinear rectification process. This framework combines diode rectification behavior with polynomial coefficients obtained from circuit simulations, enabling accurate output voltage calculation over a wide frequency band and input power range.

### 2.3. Incorporation of Impedance Mismatch via Reflection Coefficient

In RFEH systems, the impedance matching between the antenna and the rectifier directly influences the total power conversion efficiency. This matching varies with frequency in real systems and can lead to considerable reflection losses in wide-band applications. In this study, the reflection loss due to impedance matching was incorporated numerically in the model via the reflection coefficient Γ(f). The square of this coefficient |Γ(f)|^2^ represents the power ratio reflected back from the system and is given by Equation (7).

(7) $${|\Gamma \left(f\right)|}^{2}={\left|\frac{Z_{in}\left(f\right)-Z_{0}}{Z_{in}(f)+{\;Z}_{0}}\;\right|}^{2}$$

In contrast to the f^2^ and f^4^ terms, which account for the nonlinear DC components generated by the rectification process, |Γ(f)|^2^ characterizes losses due to impedance mismatch and is therefore included as an independent multiplicative factor in the model.

(8) $$V_{out}(f,P)=A(P)\;(1\;-{\;|\Gamma \left(f\right)|}^{2})\;(\alpha_{0}(P)+\alpha_{2}\left(P\right){\;f}^{2}+{\;\alpha }_{4}(P)\;f^{4})+B(P)$$

With Equation (8), the effects of rectification behavior and impedance mismatch are defined within a unified mathematical structure that depends on frequency and input power. However, in order to make this expression practically applicable, the regression coefficients included in the model must be determined so as to accurately represent the actual circuit behavior.

### 2.4. Regression Procedure and Determination of Model Coefficients

The regression coefficients used in this study were obtained from the output voltage (V_out_) curves measured at different input power levels (0, −4, −8, and −12 dBm). For each power level, the V_out_(f) data were modeled using second- and fourth-order polynomials, and the resulting coefficients (α_0_, α_2_, and α_4_) were subsequently generalized as second-order polynomial functions of the input power. The coefficients A(P) and B(P) were derived using the same regression approach. As a result, the proposed model is not limited to a single power level but remains valid over a wide range of input powers.

During model development, the harmonic content of the rectifier circuit up to the fifth harmonic was taken into account using large-signal simulations performed in the Keysight ADS environment, and the regression coefficients were derived from the corresponding simulation data. The regression calculations were carried out in MATLAB R2025a, and the accuracy of the proposed model was evaluated by comparing the modeled and simulated output voltage responses over the considered frequency band and input power range. These coefficients are defined for the designed prototype as Table 1.

The model combines impedance mismatch and diode rectification characteristics within a unified expression in terms of both frequency and input power. Thus, reflection losses resulting from antenna-rectifier matching can be analytically incorporated into the output voltage expression through the proposed model, rather than relying solely on measurement or simulation. The main distinction of the proposed model compared to its literature counterparts is that it combines the S_11_ data and the physical rectification behavior of the diode within a unified formulation. This approach improves upon classical small-signal models and provides an accurate and practical modeling framework that is sensitive to input power in wide frequency ranges; it enhances the applicability of the model for both practical applications and theoretical analyses.

## 3. Prototype Design and Experimental Setup

**Figure 2:**
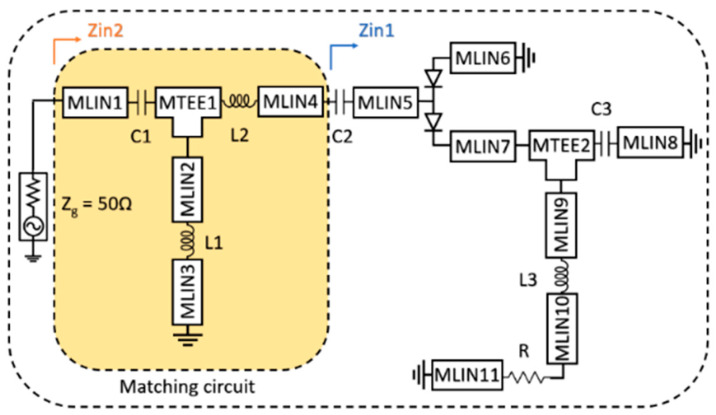
Circuit diagram of the T-type matching network and voltage doubler structure of the wide-band rectifier circuit designed for the verification of the proposed semi-analytical model.

For experimental validation, a compact and wideband RF rectifier prototype was designed and fabricated to compare the calculations of the proposed semi-analytical model with the actual circuit behavior. The prototype consists of a T-type impedance matching network followed by a voltage doubler rectifier configuration. Figure 2 illustrates the circuit topology of the wideband rectifier prototype. The lumped element values and microstrip line dimensions of the fabricated prototype used for experimental validation are summarized in Table 2.

**Figure 3:**
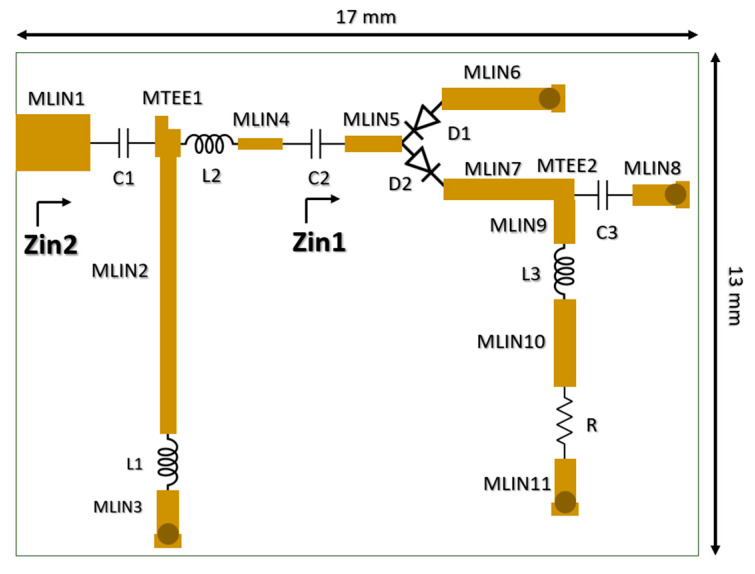
Layout of the FR4 based wide-band rectifier circuit produced for the experimental verification of the proposed semi-analytical model.

**Figure 4:**
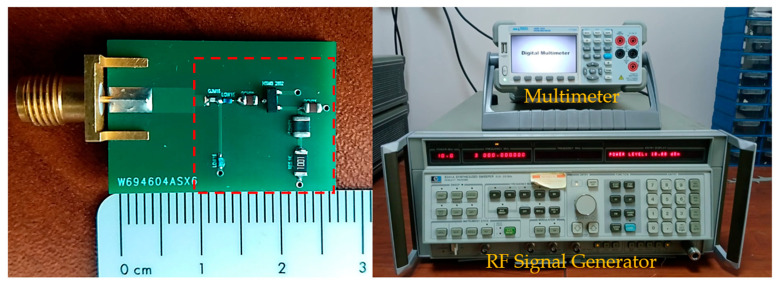
Photograph of the fabricated circuit and the devices used during the experiments.

The layout of the proposed wide-band rectifier, its photograph and the devices used during the experiments are shown in Figure 3 and Figure 4. During design and fabrication, an FR4 substrate with a dielectric constant of 4.6, a loss tangent of 0.01, and a thickness of 1.6 mm was used. Broadcom HSMS-2852 Schottky diodes were used in the circuit, and a 1 kΩ resistor was selected as the load. The prototype was manufactured with dimensions of 18 mm × 27 mm, compatible with an SMA connector, and the active circuit area was measured as 13 mm × 17 mm. Measurements were performed using an HP8341A RF signal generator, and an AAtech digital multimeter. The RF input power was swept from −12 dBm to 0 dBm over the 0.5–3 GHz frequency range, and the DC output voltage was recorded for a fixed load resistance of 1 kΩ.

## 4. Performance Evaluation of the Proposed Model

**Figure 5:**
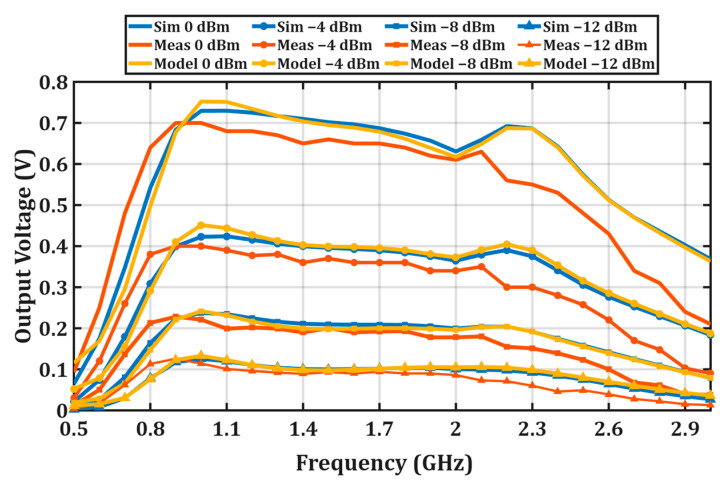
Comparison of measured, simulated, and modeled output voltage (V_out_) versus frequency at various input power levels.

Figure 5 presents a comparison of the output voltage (V_out_) responses obtained from the proposed model, simulations, and experimental measurements in the investigated frequency range. Since the regression coefficients used in the proposed model are derived from data obtained in the simulation environment, the model exhibits almost complete agreement with the simulation results for all investigated input power levels (0, −4, −8, and −12 dBm). Nevertheless, the model closely follows the experimental measurement data and successfully captures the general frequency-dependent behavior of the rectifier. The strong similarity between the output voltage profiles predicted by the model and the measured values in the same frequency ranges confirms the robustness and practical validity of the proposed modeling approach.

To quantitatively evaluate the agreement between the proposed model and the reference results, root mean square (RMS) and normalized RMS (NRMS_max_) error metrics were calculated for the 1 kΩ load condition. As summarized in Table 3, the RMS values remain below 0.02 for all investigated input power levels, while the NRMS_max_ values do not exceed 0.04. These results confirm that the proposed model provides consistent and accurate output voltage estimation across the considered frequency band and input power range.

To position the proposed approach with respect to existing RF rectifier models, Table 4 summarizes RF–DC conversion modeling studies reported in the literature, highlighting key aspects such as frequency coverage, input power dependence, and the treatment of impedance mismatch effects. As shown in the table, most existing rectifier models are developed for single-frequency applications and are typically based on narrowband operating assumptions with ideal or implicitly treated impedance matching conditions. These models primarily focus on mathematically describing the nonlinear rectification behavior of the diode or diode-connected devices and are often evaluated at a single frequency or a limited set of operating points. In contrast, the proposed model explicitly incorporates both frequency- and power-dependent behavior and accounts for frequency-dependent impedance mismatch through the S_11_ parameter within a unified formulation. This approach enables direct and consistent evaluation of rectifier performance over a wide frequency range and multiple input power levels, thereby extending the applicability of RF–DC conversion modeling beyond conventional narrowband assumptions.

## 5. Conclusions

In this study, a wide-band semi-analytical model that accurately calculations the output voltage of low-power RF rectifiers by simultaneously accounting for nonlinear diode rectification and frequency-dependent impedance mismatch was developed. The model is defined by second-order polynomial expressions for input power and frequency, providing fast and reliable performance calculations over a wide frequency band and low input power levels relevant to RF energy harvesting applications.

The proposed approach was experimentally validated using a compact rectifier prototype incorporating an FR4-based T-type impedance matching network and a voltage doubler configuration. Under the defined power and frequency range, the model calculations show very good agreement with simulation results, exceeding 95% accuracy, exceeding 95% accuracy, with root-mean-square error values remaining below 0.02 across the investigated frequency and power range.

The developed model allows rapid comparison of different impedance matching strategies by directly embedding the measured S_11_ response into the output voltage formulation, reducing the need for detailed electromagnetic simulations or multiple prototyping in the early design stages. Furthermore, its easy integration into common computing environments such as MATLAB or Excel makes it a practical and cost-effective design tool. The results demonstrate that the model is applicable to both wide-band and low-power RFEH applications and offers a robust alternative to existing approaches in the literature, particularly for wideband rectifier analysis where frequency- and power-dependent effects cannot be neglected. Future work will focus on extending the proposed formulation to different rectifier topologies, diode technologies, and antenna–rectifier interfaces, as well as investigating its applicability under broader power and frequency conditions.

## Figures and Tables

**Table 1:** Regression Coefficients of the Proposed Semi-Analytical Model as a Function of Input Power.

*A(P) = 0.6977 + 0.0795·P + 0.0027·P^2^*
*B(P) = 0.0907 + 0.0152·P + 0.0007·P^2^*
*α_0_(P) = 0.9976 − 0.0024·P − 0.0002·P^2^*
*α_2_(P) = –0.0402 − 0.0021·P − 0.0001·P^2^*
*α_4_(P) = 0.0029 + 0.0003·P − 0.0000·P^2^*

**Table 2:** Lumped Element Values and Optimized Microstrip Line Dimensions of the Validation Prototype.

Microstrip Line Dimensions				Lumped Element Values		
MLIN1 *	W:1.307	L:1.718		C1	1.8 pF	Murata GJM15
MLIN2 *	W:0.384	L:6.351		C2,3	100 pF	Murata GCM21
MLIN4 *	W:0.265	L:1.029		L1,2	6.8 nH	Murata LQW15AN
MLIN5 *	W:0.382	L:1.304		L3	1000 nH	Murata LQW21HN
MLIN3,8,9,11	W:0.500	L:1.000		R	1 kΩ	
MLIN6,7	W:0.500	L:2.500				
MLIN10	W:0.500	L:2.000				
MTEE1 *	W1:0.961	W2:0.364	W3:0.588			
MTEE2	W1:0.500	W2:0.500	W3:0.500	* Optimized microstrip lines		

**Table 3:** RMS and normalized RMS (NRMS_max_) errors between model calculations and simulation results for a 1 kΩ load.

	0 dBm	−4 dBm	−8 dBm	−12 dBm
RMS	0.02	0.01	0.01	0.01
NRMS_max_	0.03	0.03	0.03	0.04

**Table 4:** Comparison of models describing RF-to-DC conversion behavior.

Reference	Model Type	Frequency Dependance	Power Dependence	Impedance Dependence	Validation
[20] Curty et al., 2005	Analytical	Single frequency	Yes	No	Simulation
[21] Ashry et al., 2008	Analytical	Single frequency	Yes	No	Simulation
[22] Barnett et al., 2009	Analytical	Single frequency	Yes	No	Simulation
[23] Gharehbaghi et al., 2016	Analytical	Single frequency	Yes	No	Simulation + Measurement
This work	Semi-analytical	Wideband (0.5–3 GHz)	Yes	Yes (via S_11_)	Simulation + Measurement

## Data Availability

The raw data supporting the conclusions of this article will be made available by the author on request.

## Abbreviations

The following abbreviations are used in this manuscript:

RFEH	RF Energy Harvesting
WPT	Wireless Power Transfer
IoT	Internet of Things
PCE	Power Conversion Efficiency
